# Left atrial strain and diastolic function abnormalities in obese and type 2 diabetic adolescents and young adults

**DOI:** 10.1186/s12933-020-01139-9

**Published:** 2020-10-01

**Authors:** Jeremy M. Steele, Elaine M. Urbina, Wojciech M. Mazur, Philip R. Khoury, Sherif F. Nagueh, Justin T. Tretter, Tarek Alsaied

**Affiliations:** 1 The Heart Institute, Cincinnati Children’s Hospital Medical Center and the Department of Pediatrics, University of Cincinnati, Cincinnati, USA; 2 Department of Pediatrics, Section of Pediatric Cardiology, Yale University School of Medicine, PO Box 208064, New Haven, CT 06520-8064 USA; 3 The Christ Hospital Health Network Cincinnati, Cincinnati, OH USA; 4 Houston Methodist DeBakey Heart and Vascular Center, Houston, TX USA

**Keywords:** Left atrial strain, Obesity, Type 2 diabetes mellitus, Diastolic function

## Abstract

**Background:**

Adults with obesity and type 2 diabetes mellitus (T2DM) related to obesity are at increased risk of heart failure with preserved ejection fraction (HFpEF). Whether left ventricular (LV) diastolic function abnormalities related to obesity and T2DM start in adolescence and early adulthood is unknown. We non-invasively evaluated the differences seen in LV diastolic and left atrial (LA) function in adolescents and young adults with obesity and T2DM.

**Methods:**

We analyzed echocardiographic measures of LV diastolic function in patients with structurally normal hearts which were divided into 3 groups (normal weight, obese, and T2DM). Spectral and tissue Doppler and 2-D speckle tracking measurements of diastolic function were obtained. Logistic regression was performed to compare the prevalence of abnormalities in diastolic function based on the worst 25th percentile for each measure to determine the prevalence of diastolic and LA function abnormalities in obese and T2DM patients.

**Results:**

331 teenagers and young adults (median age 22.1 years) were analyzed (101 normal weight, 114 obese, 116 T2DM). Obese and T2DM group had lower E/A and higher E/e′. Obese and T2DM patients had significantly lower atrial reservoir, conduit, and booster strain and worse reservoir and conduit strain rate compared to normal patients (p < 0.001 for all measures). All patients had normal LA volumes. On multivariable analysis, conduit strain and reservoir and conduit strain rate were independently associated with having below the 25th percentile e′. Conduit strain rate was independently associated with having below the 25th percentile for mitral E/A ratio on multivariable analysis.

**Conclusions:**

Abnormal indices of LV diastolic function are detected in adolescents and young adults with obesity and T2DM. LA function and strain analysis were able to detect evidence of decreased reservoir, conduit, and booster strain in these patients although LA volume was normal. The use of LA function strain may increase our ability to detect early diastolic function abnormalities in this population.

## Introduction

Adults with obesity and type 2 diabetes mellitus (T2DM) related to obesity are at increased risk of heart failure with preserved ejection fraction (HFpEF). Obesity and type 2 diabetes (T2DM) represents an epidemic in the United States and western countries. The prevalence of obesity in adults is 38% and up to 30% in children [1]. Recently it was found that 18% of adolescents and 24% of young adults have pre-diabetes [1]. Non-invasive assessment of diastolic function remains challenging in the pediatric and young adult population as the findings of diastolic dysfunction may be the end point for long term exposure to harmful events and thus early detection is important to prevent development of heart failure symptoms [2, 3]. Left atrium (LA) maximum volume has been shown to strongly correlate with cardiovascular outcomes and has improved prognostic and diagnostic information in the assessment of LV diastolic function [3–8]. LA dilation results from chronically elevated LV filling pressures and thus is a late finding that happens in late stage I or stage II diastolic dysfunction [9]. However, recent literature suggests that LA function measures may be more sensitive early markers of LV diastolic dysfunction than LA volume data and LA strain impairment as measured by speckle tracking echocardiography may be one of the first signs of diastolic dysfunction [10–12]. The data in the pediatric and young adult population remains limited particularly as it pertains to LA dysfunction as a result of obesity and T2DM [2].

In our study we compared diastolic function and LA strain measures of adolescents and young adults who were either normal weight, obese, or diagnosed with T2DM. We hypothesized that diastolic function and LA strain function measures are impaired in patients with obesity and further impaired in T2DM as compared to the normal controls and that there are associations between LA function measures and measures of diastolic dysfunction in adolescents and young adults.

### Methods

This was a single center, retrospective review of echocardiographic measures of LV diastolic function and LA strain. The patient population used was from a previous prospective research study that examined the effects of obesity and T2DM on cardiovascular structure and function [13]. Written and informed consent were obtained from patients 18 years and older or from the patient’s guardian if they were younger than 18 years of age. The study was funded through the National Institute of Health (5R01HL105591-05). The study population was divided into three groups with T2DM, obesity (≥ 95th percentile for body mass index (BMI) but non-diabetic), and normal weight without T2DM (≤ 85th percentile for BMI). The T2DM subjects were recruited from our center’s diabetes clinic. Each diabetic subject was matched by age to one normal and one obese subject. Obese subjects had a 2-h glucose tolerance test to rule out subclinical T2DM (based on American Diabetes Association guidelines) [14]. Subjects were excluded if they had congenital heart disease (CHD), evidence of cardiomyopathy, were pregnant or if the echocardiographic image quality was thought to be inadequate to perform speckle tracking.

### Echocardiography technique

All echocardiograms were performed using the same research standardized protocol and using either a GE or Philips Sonos 5500 (Andover, MA) system with the patient in the left decubitus position. We analyzed traditional markers of diastolic dysfunction such as LA volumes and mitral inflow Doppler patterns as well as deformation analysis. Diastolic function was assessed based on the 2016 American Society of Echocardiography (ASE) guidelines [4] with the exception of tricuspid valve regurgitation jet velocity which could not be accurately assessed in the majority of our study population due to the trivial degree of regurgitation inadequate to estimate right ventricular pressure. Parasternal long-axis, short-axis, and apical 4 and 2 chamber views were recorded over 3–5 cardiac cycles with frame rates between 50 and 70 frames per second. Data collected included LA volumetric and functional data, mitral inflow and tissue Doppler. Mitral inflow velocities were obtained with pulsed wave Doppler in the apical 4-chamber view with the Doppler cursor parallel to the mitral inflow. Mitral peak E (early filling) and A (inflow with atrial contraction) waves were measured offline and an E/A ratio was calculated once imported into post-processing software (Syngo Dynamics, Siemens, Munich, Germany). Tissue Doppler velocities were acquired in the apical 4-chamber view. The peak (e′) and late velocities (a′) of mitral annular flow were recorded at both the septal and lateral annulus and were averaged. Additional diastolic variables calculated included the E/e′ using septal and lateral e′ and the average of the septal and lateral e′.

**Fig. 1:**
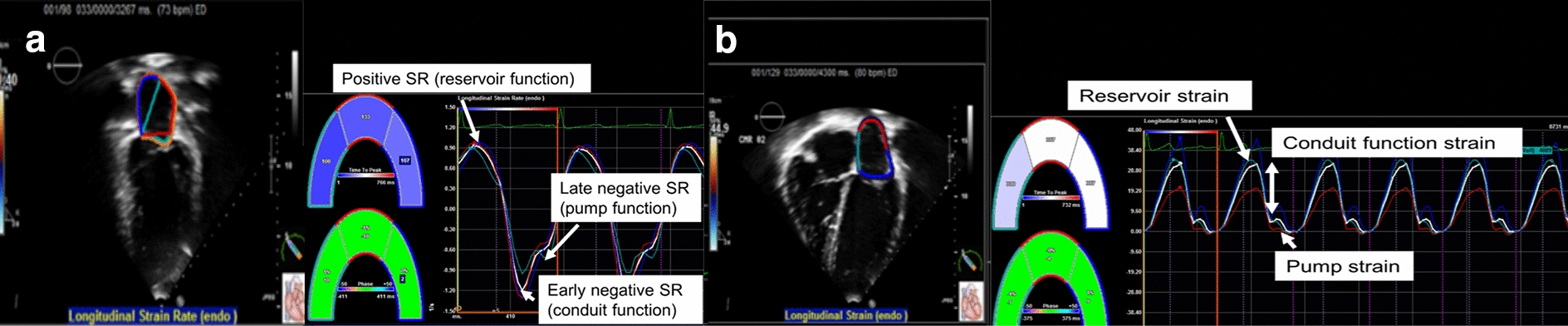
Two (**a**) and four (**b**) chamber views showing analysis and calculation of strain rate (**a**) and three components of left atrial function (**b**)

The LA functions to permit appropriate filling of the LV. There are three main functions of the LA. First, it serves as a reservoir for pulmonary venous return during ventricular systole. Second, it acts as a conduit for passive ventricular filling in early ventricular diastole, and finally as a booster pump for atrial kick at end ventricular diastole (Fig. 1) [15, 16]. LA function was studied using 2-Dimensional speckle tracking imaging (TOMTEC, Unterschleissheim, Germany). Offline and blinded analysis to the study group was performed by two pediatric cardiologists. No significant discrepancies were found by the blinded pediatric cardiologists such that a formal analysis comparing inter and intra observer variability was not performed. The apical four-chamber and two-chamber views were optimized for visualization and analysis of the LA. Patients with inadequate image quality were excluded. LA emptying fraction was calculated from echocardiography. LA areas and volumes were derived in the apical four-chamber and two-chamber views using 2-Dimensional (Simpson’s method) echocardiography. LA emptying fraction was calculated as [(maximal LA volume in ventricular systole prior to mitral valve opening − minimal LA volume after mitral valve closure)/maximal LA volume in ventricular systole just prior to mitral valve opening]. LA function measures were averaged over 3–5 cardiac cycles. The strain measurements were performed using the QRS complex (R–R gating). There are two peaks in the strain curve. The first peak corresponds to reservoir function (first peak between the R wave and the T wave) and the second to atrial contractile function (starting on the P wave); the difference between the two peaks reflects the conduit function. Positive global strain rate at the beginning of left ventricular systole reflects reservoir function. Early negative diastolic strain rate reflects conduit function; and late diastolic global strain rate reflects booster pump function (Fig. 1).

### Statistical analysis

Distributions of the data were examined for shape, and possible outliers. Bivariate scatterplots with smoothed regression lines were constructed to look for bivariate outliers, as well as for a visual representation of relationships between variables. Summary data for demographics, anthropometrics, and medication use and cardiac data were compared by study group (normal, obese, T2DM), using Fisher’s exact tests for categorical data, and Kruskal–Wallis tests for continuous data, owing to the non-parametric distribution. Bivariate correlation analyses were performed to assess association between the strain data, and the other echocardiographic data. This was done for the cohort overall, and by study group.

Differences in diastolic function between the groups were categorized as being normal or abnormal based on the 75th percentile measure of the normal group (or the 25th percentile for measures where a lower value represents dysfunction) across multiple parameters. Proportions of subjects in the obese and T2DM category with abnormal diastolic function were compared using Fisher’s exact tests. Stepwise logistic regression was performed to determine independent predictors of abnormal diastolic function as defined by the normal derived cut-points. The initial parsimonious model included demographic data and established markers of diastolic function. Logistic regression was repeated to evaluate predictors of abnormal LV function. The model included LA strain and LA size to assess the strength of contribution of these variables to LV diastolic dysfunction.

## Results

The study included 331 subjects (101 normal weight, 114 obese, 116 T2DM) with adequate echocardiographic images to determine LA function (each group started with 250 patients). Demographic data are shown in Table 1. There were significant differences between the groups pertaining to gender and race. The obese and T2DM groups had a higher percentage of females (p = 0.003) and a higher proportion of African American patients (p = 0.01). There was no significant difference in the BMI between the obese and T2DM groups and no significant difference in age among the three groups. Normal weight patients had significantly lower resting heart rates compared to obese and T2DM patients (p < 0.0001). Systolic blood pressure (SBP) and diastolic blood pressure (DBP) were higher in obese and T2DM compared to normal subjects (p < 0.0001). There were no significant differences among the groups in LV shortening fraction or ejection fraction. Indexed LV mass was higher in the obese and T2DM groups compared to the normal group (p < 0.0001).

**Table 1:** Demographics of the patient population

	Normal weight (n = 101)	Obese (n = 114)	Type 2 diabetic (n = 116)	p-value
Age (years)	21.7 (18.7, 24.6)	23.3 (20, 25.4)	22.3 (19.3, 25.6)	0.1
Male (%)	48	31	33	<0.0001
Caucasian (%)	43	31	40	<0.0001
African-American (%)	55	83	71	<0.0001
Other ethnicity (%)	3	0	5	<0.0001
BMI (kg/m^2^)	22.5 (20.7, 24.4)	36.6 (32.4, 42.5)	35.8 (30.4, 42.6)	<0.0001
Systolic blood pressure (mmHg)	110 (103, 117)	115 (109, 123)	118 (111, 127)	<0.0001
On blood pressure medication (%)	2	2	32	<0.0001
On metabolic medication (%)	0	1	58	<0.001
Duration of T2DM (years)	N/A	N/A	7.3 (5.3, 10.3)	N/A

Data is presented as medians with interquartile range (25th and 75th percentile) in parenthesis. Metabolic medication are blood glucose lower medications (i.e. Metformin)

*BMI* body mass index, *T2DM* type 2 diabetes mellitus, *N/A* not applicable

There were significant differences between the groups in several markers of LV diastolic function. Obese and T2DM patients had significantly higher average E/e′ (p < 0.0001) and lower septal (p < 0.0001) and lateral (p < 0.0001) e′ compared to the normal group. Additionally, there were significant differences in mitral inflow E/A ratios (p < 0.0001) with T2DM patients having the lowest ratio (Table 2). The direction of all these parameters indicates diastolic function abnormalities in obese and diabetic subjects.

**Table 2:** Left atrial volume and strain data and traditional diastolic function data

Variable	Normal weight (n = 101)	Obese (n = 114)	Type II diabetic (n = 116)	p-value
LA EDV (mL)	17.5 (13.6, 20.8)	21.6 (16.4, 28.7)	17.6 (13.1, 22.8)	0.001
LA ESV (mL)	44.4 (39.1, 56.1)	57.1 (46.2, 69.4)	50.7 (38.8, 60.5)	0.001
LA EF (%)	63.3 (55.7, 67.7)	63.1 (54.6, 68)	64.1 (58.6, 70.2)	0.3
LA volume (mL/m^2^)	17.4 (14.4, 19.9)	15.8 (13.1, 19.6)	13.9 (11.9, 16.9)	< 0.0001
Septal e′ (cm/s)	13.2 (11.7, 14.6)	11.6 (10.5, 13)	10.6 (9.7, 11.9)	0.0001
Septal a′ (cm/s)	6.8 (5.7, 7.6)	6.8 (5.8, 8.1)	7.4 (6.3, 8.7)	0.005
Lateral e′ (cm/s)	17.4 (15.8, 19.2)	16.1 (13.9, 17.7)	14.3 (11.7, 16.5)	0.0001
Lateral a′(cm/s)	6.1 (5.2, 7.3)	7 (5.8, 8.6)	7.3 (6.1, 9.1)	0.0001
Mitral valve E wave (cm/s)	91.6 (81.8, 100.7)	90.2 (78.8, 104.9)	92.9 (81.7, 105.9)	0.5
Mitral valve A wave (cm/s)	41.3 (34.8, 53.2)	48.4 (38.1, 58.4)	57.2 (44.2, 69.6)	0.0001
E/A (lower is worse)	2.0 (1.7, 2.5)	2.0 (1.5, 2.3)	1.6 (1.3, 2.0)	0.0001
Septal E/e′	6.8 (6.1, 7.7)	7.7 (6.6, 9.3)	8.8 (7.6, 9.8)	0.0001
Lateral E/e′	5.1 (4.6, 6.1)	5.7 (5.1, 6.5)	6.5 (5.3, 7.8)	0.0001
Average E/e′ (higher is worse)	5.9 (5.2, 6.7)	6.6 (5.8, 7.5)	7.4 (6.2, 8.7)	0.0001
Average heart rate (bpm)	63 (55, 69)	65 (58, 71)	73 (64, 80)	< 0.0001
Shortening fraction (%)	35.6 (31.4, 39.5)	36.1 (32.3, 41.3)	36.1 (32.3, 42)	0.19
LV mass (grams)	117.6 (95.4, 159)	151.5 (119.3, 188.3)	149.8 (123, 178.7)	< 0.0001
Booster function	16.6 (12.4, 20)	12.3 (9.6, 16)	13.1 (9.6, 18.4)	0.0009
Reservoir function	52.2 (44.8, 58.2)	45.1 (37.9, 53.4)	46.6 (39.7, 55.1)	0.0001
Conduit function	36.8 (30.8, 41.4)	32.8 (27.3, 37.1)	33.1 (28.7, 41.3)	0.007
Reservoir strain rate	2.0 (1.6, 2.4)	1.8 (1.3, 2.2)	1.8 (1.4, 2.2)	0.01
Conduit strain rate	− 2.6 (− 3.3, − 1.6)	− 2.2 (− 2.8, − 1.4)	− 2.1 (− 2.5, − 1.4)	0.002
Booster strain rate	− 0.2 (− 0.6, − 0.05)	− 0.4 (− 0.7, − 0.06)	− 0.4 (− 0.8, − 0.1)	0.1

Values are given as either means of the group ± standard deviation with p-values obtained using ANOVA-or medians with interquartile range (25th–75th percentile) with p-values obtained using Kruskal–Wallis

*LA* left atrium, *EDV* end diastolic volume, *ESV* end systolic volume, *EF* ejection fraction, *FAC* fractional area change

We also observed differences between the obese and T2DM groups in the prevalence of abnormal diastolic function (Table 3). Of note, 65% of T2DM subjects were in the 75th percentile or higher for average E/e′ values based on the normal control group compared to 45% of the obese group (p < 0.0001). Similar significant differences were also seen in the lateral and septal e′ values (Table 3).

**Table 3:** Comparison of abnormal distribution (based on the normal group) among groups

Variable	Worst 25th percentile (obese)	Worst 25th percentile (T2DM)	p value
E/e′	51 (45%)	75 (65%)	p < 0.0001
E/A	39 (34%)	70 (60%)	p < 0.0001
Reservoir strain	55 (48%)	49 (42%)	p = 0.001
Conduit strain	47 (41%)	45 (39%)	p = 0.024
Reservoir strain rate	40 (35%)	43 (37%)	p = 0.305
Conduit strain rate	35 (31%)	36 (31%)	p = 0.649
LAD/m^2^	34 (30%)	37 (32%)	p = 0.451

Data is presented as number of patients with percentage in parenthesis. P value represents comparison of obese and T2DM

*LAD* Left atrial dimension

### LA function differences between the groups

Obese and T2DM had significantly lower reservoir (p < 0.0001), conduit (p = 0.007) and booster strain (p = 0.0002) compared to normal subjects (Table 2).

Strain rate analysis showed a significantly lower reservoir strain rate in obese and T2DM subjects (p = 0.01). The conduit strain rate was also significantly worse (less negative) in obese and T2DM subjects (p = 0.001). There were no significant differences among the groups in booster strain rate.

There were no differences among the groups in left atrial dimension indexed to body surface area (p = 0.2). However, indexed left atrial volume to body surface area was significantly lower in the obese and T2DM groups compared to the normal group (p < 0.0001) due to the larger body surface area in obese and T2DM groups. There was no significant difference among the groups in atrial emptying fraction (p = 0.3).

### Univariate associations of left atrial strain with other echocardiographic measures

Correlation analysis showed that higher LV mass was associated with lower reservoir and conduit strain (p < 0.0001), reservoir strain rate (p = 0.003), and worse conduit strain rate (p = 0.002). A higher indexed LA volume was associated with lower conduit strain and worse reservoir strain rate (Additional file 1).

Reservoir and conduit strain were positively associated with lateral and septal e′, and mitral E/A ratio. Conduit strain rate was negatively associated with lateral and septal e′, and mitral E/A ratio, while it was positively associated with E/e′ ratio. This indicates that a higher conduit strain rate (less negative, worse) correlated to a lower e′ and thus a higher E/e′ ratio (Additional file 1).

### Predictors of differences in diastolic function

Multivariable analysis showed that higher conduit strain rate (less negative) was a significant predictor of being in the worst 25th percentile of mitral E/A (odds ratio (OR), 2.18; confidence interval (CI) 1.57–3.03 per unit increase in strain rate, p < 0.0001). Additionally, a lower reservoir strain rate was a significant predictor of being in the worst 25th percentile for septal e′ (OR, 2.15; CI 1.24–3.72 per unit decrease in strain, p = 0.006). Importantly, indexed LA volume and LA dimension were not predictors of diastolic dysfunction (Table 4).

**Table 4:** Risk factors predicting the worst 25th percentile of diastolic dysfunction measures

Factor	OR for worst 25th percentile E/e′	OR worst 25th percentile septal e′	OR worst 25th percentile lateral e′	OR worst 25th percentile mitral E/A
Female gender		3.07 (1.71–5.53)		2.23 (1.20–4.12)
T2DM as reference	1	1	1	1
Normal	0.32 (0.16–0.62)	0.21 (0.11–0.41)	0.19 (0.09–0.35)	0.34 (0.16–0.70)
Obese	0.56 (0.30–1.03)	0.45 (0.25–0.81)	0.38 (0.22–0.68)	0.34 (0.18–0.64)
SBP	1.03 (1.01–1.06)	1.03 (1.01–1.06)		
DBP			1.04 (1.01–1.07)	1.05 (1.02–1.08)
LAV/m^2^	0.93 (0.89–0.99)			
LAD/m^2^		2.8 (1.19–6.58)		
Average conduit strain (per 1 unit decrease)		0.96 (0.93–0.99)		
Average conduit strain rate (per 1 unit increase)			1.58 (1.17–2.12)	2.18 (1.57–3.03)
Average reservoir strain rate (per 1 unit decrease)		2.15 (1.24–3.72)		

## Discussion

Adolescents and young adults with obesity and T2DM had higher E/e’ and lower E/A ratios compared to the normal group despite having no LA enlargement. Obese and T2DM subjects were also found to have significantly worse LA function strain of all three LA functional components and significantly worse reservoir and conduit strain rates compared to controls. Atrial strain measures associated with diastolic function abnormalities in obese and T2DM groups.

Although the majority of these diastolic function indices are still within normal range based on the diastolic function guidelines [4], our study is novel in demonstrating significant differences in diastolic function starting at a young age. This may imply a trend for further and quicker worsening of diastolic function of the obese and T2DM group if they were followed for a longer period. This theory is supported by studies which have shown childhood adiposity to be independently associated with structural cardiac disturbances [17], as well as abnormalities in LA function in patients with long-standing metabolic syndrome [18]. Though not specifically focusing on LA function other studies have shown that adolescents with T2DM have lower E/A ratios [19]. In addition, when we used the worst 25th percentile cut point for diastolic function based on the normal subjects in this cohort, a high proportion of obese and T2DM subjects were in the group with abnormal diastolic function. Our study also showed that LA diameter is an insensitive measure for diastolic dysfunction. Our multivariable analysis did not show LA size to be a predictor of diastolic function abnormalities in obese and T2DM patients. Furthermore, the indexed LA volume was significantly larger in the normal group compared to the T2DM and obese groups. This is likely due to the higher body surface area in the latter groups. Thus, LA volume cannot be used to detect early diastolic dysfunction in obese adolescents and young adults as LA enlargement is likely a late sign and may not be accurately perceived when indexed to a relatively large body size.

Additionally, we show that LA strain measures have an independent association with diastolic function abnormalities on multivariate analysis. Obese and T2DM patients have a worse reservoir, conduit and booster strain and worse reservoir and conduit strain rate. This is not surprising since studies in adults have shown that LA strain becomes abnormal over a decade before the LA becomes dilated [20].

Epidemiological analysis has linked obesity and T2DM as a risk factor for atrial fibrillation, though an exact mechanism is still unclear, it could be related to atrial function abnormalities [21]. In adults reduced LA reservoir and conduit function as well as changes in booster function have been used to predict the onset of paroxysmal atrial fibrillation [22, 23]. Progression to chronic atrial fibrillation has also been shown to be closely related to LA reservoir strain [24]. Similarly, measures of LA function and strain have been shown to improve following ablation for atrial fibrillation with return of normal sinus rhythm [25]. Furthermore, LA reservoir strain has been shown to be a better predictor of elevated left ventricle end-diastolic pressure than average E/e′ ratios [26, 27]. In patients with normal LA size, studies have shown that reduced reservoir strain was a predictor of developing NYHA class II and IV symptoms within 2 years and had good correlation with pulmonary capillary wedge pressure [27, 28]. Despite these benefits of measuring LA functional parameters many practitioners may be more comfortable using LV GLS as a reliable method of LV diastolic function. We are not suggesting LA function measurements should replace LV GLS, but rather that LA function measurements act as a complementary measurement, which may be able to detect LV diastolic dysfunction earlier than LV GLS.

The Strong Heart Study showed that the presence of diabetes mellitus alone is a risk factor for the development of abnormal atrial function [29]. Studies by Di Salvo et al. showed that obesity (even with normal blood pressure) in the pre and early pubertal children was associated with abnormal atrial strain [30]. Although the majority of the patients in our study had normal blood pressure, early-onset hypertension has been associated with adverse LA remodeling [31]. Similar studies conducted in adult patients followed longitudinally concluded that obesity is associated with impaired reservoir and conduit function [32], and that obesity, regardless of the presence of T2DM is associated with abnormal LV global longitudinal strain [33]. In our study, we showed that abnormal diastolic function measures and LA function were already present in many of the obese and T2DM patients at a young age. Therefore, one could postulate that adolescents and young adults with obesity and T2DM are at risk to develop abnormal left atrial function earlier than normal weight patients. Regardless of etiology, there appears to be a common pathway to atrial dysfunction. In early stages there is a decrease in reservoir and conduit function with a compensatory increase in contractile (booster pump) function [5, 8, 31]. Our study also suggests this to be true as the booster strain rate was not different between the groups, possibly because of their young age. In the more advanced phase of diastolic dysfunction which often coincides with the onset of heart failure symptoms, there is additionally a decrease in contractile function resulting in lower booster strain with further decrease in reservoir and conduit function [32].

An important question is whether T2DM in this population adds deleterious role in diastolic function compared to obesity. In our study, the T2DM patients had significantly higher E/e′ and lower E/A ratios compared to the obese group and as mentioned were more likely to be in the percentile group trending towards abnormal diastolic function. However, blood pressure, LV mass, and atrial strain and strain rates were not different between the two groups. This may indicate that at this early age T2DM does not compound the cardiac remodeling associated with obesity, but further studies would be needed to better assess this.

One factor that may improve diastolic dysfunction in T2DM is the use of sodium-glucose cotransporter 2 inhibitors (SGLT2i). Multiple studies have shown improvement in LV diastolic function within 3 months of therapy [34, 35]. These studies assessed the effect of these medications and found improvement in E/e′ in patients prescribed SGLT2i. LA function parameters were not specifically assessed in these studies but would be an interesting focus of future analysis.

A major concern for using LA function as a reliable assessment of diastolic function is the inter and intra observer variability. It is important to note, that echocardiography is a reliable method to assess LA functional parameters [36]. In our study the initial LA measurements were reanalyzed by multiple imaging trained pediatric cardiologists with no significant discrepancies.

These aforementioned studies are important for children and young adults such as those in our study population, who currently are asymptomatic but considered at higher risk for development of atrial fibrillation and diastolic heart failure. Clinically, the implications of our study are relevant, even in the absence of longitudinal follow up data due to the noted literature of the negative effects of obesity and T2DM on diastolic function in adults [18, 19, 32, 33]. Thus, pediatric and adult cardiologists can expect progression to abnormal diastolic function and LA functional data may aid efforts for early detection of diastolic function abnormalities in this young patient population. From a practical standpoint, this information may help inform the patients and families about the adverse effects of obesity on the cardiovascular system.

### Limitations

There were several limitations to our study. Firstly, this was a single center retrospective study. Secondly, there is a lack of outcome data owing to the grant for the initial study ending. The majority of the patients in this study had normal diastolic function, despite differences between the groups and this may be perceived as a limitation of clinical impact, however, given the young age of the patients in this study, the trend towards abnormality is concerning that many of these patients will progress to abnormal diastolic function at a young age. We also could not include tricuspid regurgitation velocity, a known important marker of diastolic function in our assessment as most of our subjects had trivial tricuspid regurgitation insufficient to assess right ventricular pressure. An important question we could not answer from this data, is whether the LA function and strain became abnormal prior to the mitral and tissue Doppler markers, as that would support our theory that LA strain may be the first marker of diastolic dysfunction in this patient population. Additionally, the causes of HFpEF are multifactorial including age and additional comorbidities which limits our abilities to make definitive conclusions. Finally, although the initial studies were evaluated by two pediatric cardiologists, we did not perform a formal statistical analysis for reproducibility in our study as measuring atrial strain has been validated by previous studies [37].

## Conclusions

Abnormal diastolic function measures were detected in adolescents and young adults with obesity and T2DM. LA function and strain analysis were able to detect evidence of decreased reservoir, conduit, and booster atrial function in these subjects although LA volume was normal. LA function measures were associated with diastolic function measures and predicted subjects in the worst 25th percentile of diastolic function measures. The use of LA function strain may provide early detection of diastolic function abnormalities.

## Supplementary information

**Additional file 1: Table S1.** Correlations between traditional diastolic function measurements and left atrial strain measurements of the entire cohort. **Table S2.** Correlations between traditional diastolic function measurements and left atrial strain measurements of the normal weight patients (n = 101). **Table S3.** Correlations between traditional diastolic function measurements and left atrial strain measurements of the obese patients (n = 114). **Table S4.** Correlations between traditional diastolic function measurements and left atrial strain measurements of T2DM patients (n = 116).

## Data Availability

All datasets used and analyzed during this study are available from the corresponding or senior author on reasonable request.

## Abbreviations

T2DM: Type 2 diabetes mellitus
HFpEF: Heart failure with preserved ejection fraction
LV: Left ventricle
LA: Left atrium
BMI: Body mass index
CHD: Congenital heart disease
GE: General electric
ASE: American society of echocardiography
CI: Confidence interval
OR: Odds ratio
GLS: Global longitudinal strain
