# Supplementary material for: Left atrial strain and diastolic function abnormalities in obese and type 2 diabetic adolescents and young adults
Source: Cardiovasc Diabetol. 2020 Oct 1;19:163. doi: 10.1186/s12933-020-01139-9 (PMC7531140; doi:10.1186/s12933-020-01139-9)
Supplement: Supplementary file 1 — Additional file 1: Table S1. Correlations between traditional diastolic function measurements and left atrial strain measurements of the entire cohort. Table S2. Correlations between traditional diastolic function measurements and left atrial strain measurements of the normal weight patients (n = 101). Table S3. Correlations between traditional diastolic function measurements and left atrial strain measurements of the obese patients (n = 114). Table S4. Correlations between traditional diastolic function measurements and left atrial strain measurements of T2DM patients (n = 116). [file 12933_2020_1139_MOESM1_ESM.pdf]

Variable	LVM/m^2	LAV/m^2	Septal e'	Lateral e'	Average E/e'	E/A
Reservoir Strain	-0.26<br>p<0.0001	-0.04<br>p=0.46	0.13<br>p=0.01	0.22<br>p<0.0001	-0.04<br>p=0.43	0.11<br>p=0.05
Conduit Strain	-0.25<br>p<0.0001	-0.11<br>p=0.04	0.17<br>p=0.001	0.19<br>p=0.0007	-0.02<br>p=0.63	0.17<br>p=0.002
Reservoir Strain Rate	-0.17<br>P=0.003	-0.19<br>p=0.0006	0.005<br>p=0.92	0.15<br>p=0.006	-0.09<br>p=0.11	0.12<br>p=0.02
Conduit Strain Rate	0.17<br>P=0.002	0.11<br>p=0.05	-0.15<br>p=0.009	-0.28<br>p<0.0001	0.13<br>p=0.02	-0.3<br>p<0.0001

Pearson correlation analysis (first line) and p-value (second line). LVM/m^2 - Left ventricular mass indexed to BSA. LAV/m^2 – Left atrial volume indexed to BSA

Variable	LVM/m^2	LAV/m^2	Septal e'	Lateral e'	Average E/e'	E/A
Reservoir Strain	-0.24<br>P=0.01	-0.09<br>p=0.35	0.11<br>p=0.26	0.19<br>P=0.05	-0.004<br>p=0.96	0.12<br>p=0.23
Conduit Strain	-0.19<br>P=0.06	-0.18<br>p=0.07	0.19<br>p=0.05	0.18<br>p=0.09	0.02<br>p=0.83	0.18<br>p=0.07
Reservoir Strain Rate	-0.003<br>P=0.98	-0.21<br>p=0.04	-0.01<br>p=0.88	0.15<br>p=0.13	-0.01<br>p=0.85	0.02<br>p=0.85
Conduit Strain Rate	-0.04<br>P=0.69	0.13<br>p=0.17	-0.13<br>p=0.18	-0.14<br>P=0.15	0.01<br>p=0.88	-0.19<br>P=0.05

Pearson correlation analysis (first line) and p-value (second line). LVM/m^2 - Left ventricular mass indexed to BSA. LAV/m^2 – Left atrial volume indexed to BSA

Variable	LVM/m^2	LAV/m^2	Septal e’	Lateral e’	Average E/e’	E/A
Reservoir Strain	-0.12<br>P=0.23	-0.12<br>p=0.21	0.03<br>p=0.72	0.20<br>P=0.03	-0.0001<br>p=0.99	0.11<br>p=0.24
Conduit Strain	-0.13<br>P=0.02	-0.17<br>p=0.07	0.13<br>p=0.18	0.25<br>p=0.009	0.004<br>p=0.95	0.23<br>p=0.01
Reservoir Strain Rate	-0.18<br>P=0.05	-0.22<br>p=0.02	-0.11<br>p=0.25	0.28<br>p=0.003	-0.27<br>p=0.006	0.13<br>p=0.19
Conduit Strain Rate	0.25<br>P=0.01	0.16<br>p=0.09	0.02<br>p=0.84	-0.39<br>P<0.0001	0.21<br>p=0.03	-0.33<br>P=0.0005

Pearson correlation analysis (first line) and p-value (second line). LVM/m^2 - Left ventricular mass indexed to BSA. LAV/m^2 – Left atrial volume indexed to BSA

Variable	LVM/m^2	LAV/m^2	Septal e’	Lateral e’	Average E/e’	E/A
Reservoir Strain	-0.25<br>P=0.009	-0.02<br>p=0.79	0.05<br>p=0.58	0.15<br>P=0.10	0.01<br>p=0.86	-0.01<br>p=0.90
Conduit Strain	-0.19<br>P=0.04	-0.06<br>p=0.49	0.13<br>p=0.15	0.10<br>p=0.26	-0.01<br>p=0.86	0.04<br>p=0.62
Reservoir Strain Rate	-0.13<br>P=0.19	-0.30<br>p=0.001	-0.10<br>p=0.29	-0.11<br>p=0.21	0.10<br>p=0.26	0.10<br>p=0.26
Conduit Strain Rate	0.11<br>P=0.27	0.23<br>p=0.01	-0.06<br>p=0.49	-0.17<br>P=0.07	-0.23<br>p=0.81	-0.26<br>P=0.004

Pearson correlation analysis (first line) and p-value (second line). LVM/m^2 - Left ventricular mass indexed to BSA. LAV/m^2 – Left atrial volume indexed to BSA
